# PPAR-*γ* Signaling Crosstalk in Mesenchymal Stem Cells

**DOI:** 10.1155/2010/341671

**Published:** 2010-07-26

**Authors:** Ichiro Takada, Alexander P. Kouzmenko, Shigeaki Kato

**Affiliations:** ^1^Department of Microbiology and Immunology, School of Medicine, Keio University, 35 Shinano-machi, Shinjuku-ku, Tokyo, 160-8582, Japan; ^2^College of Science & General Studies, Alfaisal University, P.O. Box 50927, Riyadh 11533, Saudi Arabia; ^3^Institute of Molecular and Cellular Biosciences, University of Tokyo, Yayoi 1-1-1, Bunkyo-ku, Tokyo 113-0032, Japan

## Abstract

Peroxisome proliferator-activated receptor-gamma (PPAR-*γ*) is a member of the nuclear receptor (NR) superfamily of ligand-activated transcriptional factors. Among other functions, PPAR-*γ* acts as a key regulator of the adipogenesis. Since several cytokines (IL-1, TNF-*α*, TGF-*β*) had been known to inhibit adipocyte differentiation in mesenchymal stem cells (MSCs), we examined the effect of these cytokines on the transactivation function of PPAR-*γ*. We found that the TNF-*α*/IL-1-activated TAK1/TAB1/NIK (NF*κ*B-inducible kinase) signaling cascade inhibited both the adipogenesis and Tro-induced transactivation by PPAR-*γ* by blocking the receptor binding to the cognate DNA response elements. Furthermore, it has been shown that the noncanonical Wnts are expressed in MSCs and that Wnt-5a was capable to inhibit transactivation by PPAR-*γ*. Treatment with Wnt5a-activated NLK (nemo-like kinase) induced physical association of the endogenous NLK and H3K9 histone methyltransferase (SETDB1) protein complexes with PPAR-*γ*. This resulted in histoneH3K9 tri-methylation at PPAR-*γ* target gene promoters. Overall, our data show that cytokines and noncanonical Wnts play a crucial role in modulation of PPAR-*γ* regulatory function in its target cells and tissues.

## 1. Introduction

Peroxisome proliferator-activated receptor-gamma (PPAR-*γ*) belongs to the nuclear receptor (NR) superfamily and regulates target gene mRNA expression in the ligand-dependent manner [1]. Similar to most known NRs, PPAR-*γ* contains distinct domains for binding the DNA (DBD), ligand (LBD), and various cofactor complexes. The structure of PPAR-*γ* LBD consists of 12 *α*-helices and 4 *β*-sheets [2]. 

For ligand-dependent transcriptional control by PPAR-*γ*, several distinct classes of transcriptional coregulators/coregulator complexes are indispensable in addition to basic transcription machinery to reorganize chromatin state at the genomic target loci [1, 3]. Transcriptional coregulators for NRs can be divided into two classes in regard to the mechanisms of chromatin reorganization. One class consists of histone modifying enzymes that reversibly modify the N-terminal tails of nucleosomal histone proteins [4, 5]. For example, acetylation and methylation at histone H3K4 and H3K36 are chromatin activating modifications and support transcriptional up-regulation by NRs [6, 7]. In contrast, transcriptional repression by NRs is coupled with inactivating modifications like deacetylation and methylation at histone H3K9 and H3K27 [8]. Accordingly, cognate histone modifying enzymes serve as NR coregulators. 

The other class of transcriptional coregulators includes chromatin remodeling factors that directly reorganize nucleosomal arrays using ATP hydrolysis as a source of energy [9, 10]. Chromatin remodelers function as multi-subunit complexes and include ATPase catalytic subunits. Four distinct types of chromatin remodeling complexes (SWI/SNF, ISWF, WINAC, and NURD) have been so far identified as transcriptional coregulators of NRs [11, 12]. 

**Figure 1:**
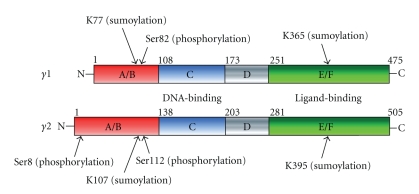
Structure and posttranslational modifications of PPAR-*γ*1, - *γ*2 proteins. Although PPAR-*γ* was ubiquitinated, lysine residues are not determined [18].

Besides ligand dependency, various signaling pathways modulate the ligand-dependent transactivation function of NRs. For example, phosphorylation in the N-terminal region of estrogen receptor alpha (ER-*α*) by certain pathway-activated protein kinases enhances the transactivation function of ER-*α* [13]. The transcriptional activity of PPAR-*γ* is also modulated through positive and negative crosstalk with other signaling pathways [14]. The molecular mechanisms of the crosstalk include direct and indirect associations of PPAR-*γ* with intracellular signal transducers or transcriptional factors as well as covalent modifications of PPAR-*γ* protein, such as phosphorylation by signal-dependent protein kinases [15] or sumoylation by UBC9 [16]. Phosphorylation of PPAR-*γ* in the N-terminal domain suppresses the transactivation function of PPAR-*γ* by reducing affinity for PPAR-*γ* ligands [17], whereas ligand-dependent sumoylation of PPAR-*γ* represses the NF-*κ*B activation and antagonizes inflammatory responses [16]. These clearly indicate that modifications in the PPAR-*γ* molecule play a pivotal role in modulation of its physiological action (Figure 1).

## 2. Signaling Crosstalk between PPAR-*γ* and Cytokines in MSCs

 Mesenchymal stem cells (MSCs) derived from various adult tissues have the potential to differentiate into different lineages, including osteoblasts, chondrocytes, adipocytes, or myocytes [19–21]. Reflecting such pluripotency, a number of regulators involved in the control of MSC differentiation have been identified and characterized [19]. Bone morphogenetic protein (BMP) signaling molecules (particularly BMP-2, -4, -6, and -7) act as major osteogenic inducers and may also influence adipocyte differentiation [22] through induction of PPAR-*γ* corepressor, TAZ [23]. Recently, the hedgehog signaling has been shown to inhibit adipogenesis and induce osteoblastogenesis [24].

 Since several cytokines (IL-1, TNF-*α*, TGF-*β*) inhibit adipocyte differentiation in MSC, we examined the effect of their signaling on the transactivation function of PPAR-*γ*. Treatment with TNF-*α* or IL-1 inhibited Tro-induced transcriptional activity of PPAR-*γ*. Interestingly, treatment with both Tro and cytokine (IL-1 or TNF-*α*) induced osteoblastogenesis in ST2 cells. Thus, cytokines and activated PPAR-*γ* appeared to stimulate cytodifferentiation of bone marrow progenitor cells into osteoblasts, in addition to cytokine-dependent interference with adipocyte differentiation. Since TNF-*α* and IL-1 are known to activate the NF-*κ*B in the nucleus, and the nuclear NF-*κ*B is indispensable for osteoclastogenesis from heamatopoetic stem cells, these cytokines appear to be physiologically important for the mesenchymal stem cell fate decision. We therefore studied effects of downstream mediators of the TNF*α*/IL-1 signaling on the MSC differentiation [14].

In ST2 cells, the TNF-*α*/IL-1-activated TAK1/TAB1/NIK (NF*κ*B-inducible kinase) signaling cascade inhibited both the adipogenesis and Tro-induced transactivation by PPAR-*γ*. Though it was previously reported that phosphorylation of PPAR-*γ* by MAP kinase resulted in repression of the PPAR-*γ* function [15], we showed that TNF-*α*/IL-1-induced inhibition of PPAR-*γ* did not involve its phosphorylation by the NIK. 

**Figure 2:**
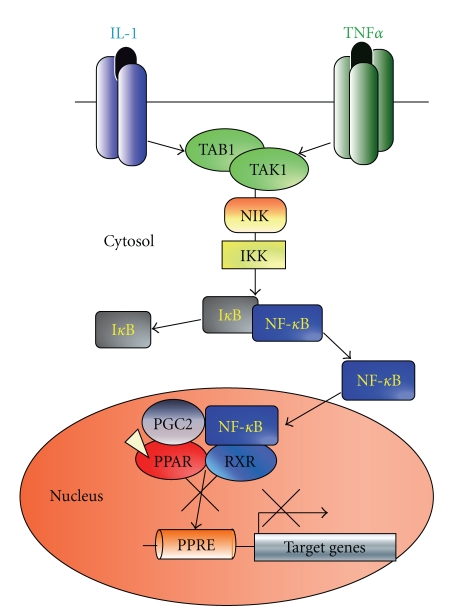
Schema of the proposed molecular mechanism of adipogenesis inhibition by TNF-*α* and IL-1 through suppression of PPAR-*γ* function by NF-*κ*B activated via the NIK-TAK1/TAB1-mediated cascade.

Consistent with suppression of the PPAR-*γ*-dependent luciferase reporter gene activity, the activated TAK1/TAB1/NIK was found to suppress the Tro-induced expression of endogenous PPAR-*γ* target genes. We found that treatment with these cytokines or ectopic expression of some of their downstream mediators blocked binding of PPAR-*γ* to its response element DNA sequences (PPRE) in the target gene promoters (Cbl-associated protein, CAP). CAP is a signaling protein that interacts with both c-Cbl and the insulin receptor that may be involved in the specific insulin-stimulated tyrosine phosphorylation of c-Cbl [25, 26]. Next, we have shown that the TAK1/TAB1/NIK pathway-activated NF-*κ*B blocks the DNA binding of PPAR-*γ* at the PPRE. Together with the previous reports that agonist-activated PPAR-*γ* inhibits DNA binding by NF-*κ*B [27], it appears that an association of ligand-activated PPAR-*γ* with nuclear NF-*κ*B results in a complex incapable to interact with DNA at either corresponding binding sites (Figure 2). 

 Thus, we presume that TNF-*α*/IL-1 triggers activation of NF-*κ*B through the TAK1/TAB1/NIK axis, leading to a physical association between PPAR-*γ* and NF-*κ*B thereby inhibiting the ligand-dependent PPAR-*γ* transactivation. Since PPAR-*γ* is a prime regulator of adipogenesis, suppression of the PPAR-*γ* function may inhibit adipogenesis and consequently, shift the bone marrow cell fate decision towards the osteoblastogenesis [14].

## 3. Noncanonical Wnt Signaling Induces Osteoblastogenesis through Transrepression of PPAR-*γ* by Histone Methyltransferase Complex

 Our recent studies of the effects of Wnts on the osteoblastogenesis and adipogenesis have shown that Wnt signaling may directly regulate the transactivation function of PPAR-*γ* in the MSCs [28]. Several frizzled receptors and Wnt ligands have been found expressed at significant levels in the ST2 cells and in mouse bone marrow cell primary culture. Interestingly, noncannonical Wnt ligand (Wnt-5a) and receptors (Frizzled-2 and -5) were found to be expressed in these cells at particular high levels [28]. While Wnt-3a, a canonical Wnt ligand, did not affect transactivation function of Tro-induced PPAR-*γ*, noncanonical Wnt-5a was capable to repress activation by PPAR-*γ* recombinant and endogenous PPAR-*γ* target gene promoters. We then explored an ability of downstream mediators of the Wnt-5a signaling to repress PPAR-*γ* and determined that CaMKII-TAK1/TAB2-NLK axis members were potent inhibitors of the receptor. This was consistent with reports that NLK-deficient mice exhibited increased adipocyte concentration in the bone marrow [29]. 

As the NLK acts as a downstream mediator in the Wnt-5a signaling pathway, we explored molecular basis of the transrepressive effects of NLK on the PPAR-*γ* transcriptional function. Since tricostatine A, an inhibitor of a wide range of HDACs, was unable to reverse the NLK-mediated suppression of PPAR-*γ* function, this opened a question about possible involvement of other inactivating histone modifying enzymes. NLK-containing protein complexes were biochemically purified from nuclear extracts of KCl-treated HeLa cells expressing FLAG-tagged NLK [9, 30] and a distinct NLK-nuclear protein complex with a molecular weight of around 400–500 kDa was isolated and analysed [28, 31]. In this complex, a 170 kDa component was identified as a SETDB1, a transcription inhibiting histone lysine-methyltransferase (HKMT) that methylates histone H3 at K9 [32, 33]. Importantly, in ST2 cells, treatment with Wnt5a induced a physical association of endogenous NLK-SETDB1 protein complexes with PPAR-*γ*. 

**Figure 3:**
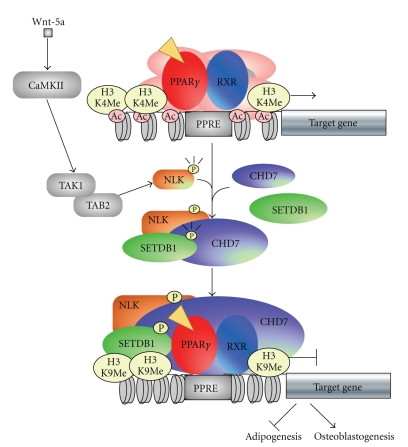
Schematic model of crosstalk between PPAR-*γ* and Wnt-5a signaling in MSC. NLK activated by the Wnt5a signaling pathway phosphorylates SETDB1 and forms a complex with PPAR-*γ*/RXR and chromodomain containing protein 7 (CHD7).

ChIP analysis of endogenous transcriptional factors and histone modifications at the PPAR-*γ* response element (PPRE) in the aP2 gene promoter [34] has shown that treatment with Tro induced recruitment of known PPAR-*γ* coactivator SRC-1. However, simultaneous treatment with Wnt-5a and Tro induced recruitment of NLK and SETDB1 at the PPRE region. Consistently, an increase in histone H3 di- and tri-methylation at K9 was observed together with histone hypoacetylation. Such coordinated chromatin silencing histone modifications at the PPAR-*γ* target genes were more prominent after a 7-day treatment with Wnt-5a that was long enough to induce the osteoblastogenesis. Furthermore, an ectopic expression of either NLK or SETDB1 in the presence of Tro was potent to induce the osteoblastogenesis and inhibit the adipogenesis, whereas a knockdown of either NLK or SETDB1 potentiated the Tro-induced adipogenesis even in the presence of Wnt-5a. Thus, we have shown that Wnt-5a induces the osteoblastogenesis through attenuating the PPAR-*γ*-induced adipogenesis in the bone marrow MSC (Figure 3). 

Upon Wnt-5a-induced activation of the noncanonical Wnt signaling, the SETDB1 HKMT forms a complex with phosphorylated NLK. This NLK/SETDB1 complex associates with PPAR-*γ* and methylates H3-K9 at the PPAR-*γ* target gene promoters leading to their transcriptional silencing. Interestingly, the NLK also suppresses the transactivation function of the A-Myb through histone methylation [35], suggesting that the NLK might control gene expression by histone modification through recruitment of SETDB1. 

The noncanonical Wnt-5a ligand regulates MSC differentiation through the CaMKII-TAK1/TAB2-NLK signaling cascade that is distinct from the canonical Wnt pathway, which is mediated by the *β*-catenin/TCF signal transduction. Several recent reports have demonstrated that the canonical Wnt pathway mediated by LRP5/*β*-catenin is also indispensable for the osteoblastogenesis [36–38]. Hence, both the canonical and noncanonical Wnt pathways are considered to support the osteoblastogenesis in the bone marrow mesenchymal cells. However, only the noncanonical Wnt signaling appears to impair the PPAR-*γ*-inducible adipogenesis and switch the MSC differentiation into the osteoblastic lineage.

## 4. Conclusion

 In summary, IL-1, TNF-*α*, and noncanonical Wnt signaling pathways suppress the PPAR-*γ* function in MSCs and thus, are capable to influence stem cell fate [39]. Interestingly, molecular mechanism of suppression of the PPAR-*γ* transcriptional activity by the IL-1 and TNF-*α* is different from that induced by the noncanonical Wnt ligands. IL-1 or TNF-*α*-activated NF-*κ*B inhibits the DNA binding capacity of the receptor, while Wnt5a-activated NLK promotes PPAR-*γ*/SETDB1 complex formation leading to silencing epigenetic chromatin modifications at the PPRE. Recent studies show that PPAR-*γ* also plays pivotal roles in other cells and tissues, such as osteoclasts [40], kidney cells [41], and macrophages [27]. This opens questions about the existence of other mechanisms of modulations of the PPAR-*γ* physiological activity specific for these types of differentiated cells that may be different from those in stem cells.
